# ESR1 analysis of liquid biopsy in breast cancer, one-year routine experience of an Italian clinical referral center

**DOI:** 10.1016/j.jlb.2025.100331

**Published:** 2025-10-08

**Authors:** Thais Maloberti, Laura Poppi, Giulia Ciccimara, Sara Coluccelli, Floriana Jessica Di Paola, Giulia Calafato, Viviana Sanza, Elisa Gruppioni, Annalisa Altimari, Sara Quercia, Alessandra Bernardi, Claudio Zamagni, Roberta Minari, Antonio De Leo, Giovanni Tallini, Dario de Biase

**Affiliations:** a Solid Tumor Molecular Pathology Laboratory, IRCCS Azienda Ospedaliero-Universitaria di Bologna, Bologna, Italy; b Biobank of Research, IRCCS Azienda Ospedaliero-Universitaria di Bologna, Bologna, 40138, Italy; c Division of Oncology, IRCCS Azienda Ospedaliero-Universitaria di Bologna, Bologna, Italy; d Medical Oncology Unit, University Hospital of Parma, Parma, Italy; e Department of Medical and Surgical Sciences (DIMEC), University of Bologna, Bologna, Italy; f Department of Pharmacy and Biotechnology (FaBiT), University of Bologna, Bologna, Italy

**Keywords:** Liquid biopsy, ESR1, Breast cancer, NGS, ctDNA

## Abstract

**Background:**

Activating mutations in the *ESR1* gene are a known mechanism of secondary resistance to endocrine therapy in metastatic estrogen receptor-positive (ER+)/human epidermal growth factor receptor 2-negative (HER2−) breast cancers. Liquid biopsy has become a non-invasive tool for molecularly characterizing these neoplasms and allows for dynamic monitoring through the analysis of circulating tumor DNA (ctDNA).

**Methods:**

We analyzed 161 plasma samples from patients with metastatic ER+/HER2− breast cancer who had previously undergone treatment with endocrine therapy and CDK4/6 inhibitors. *ESR1* mutation analysis was performed using two NGS panels: Oncomine™ Breast cfDNA Assay v2 (n = 102) and Oncomine™ Precision Assay GX (n = 59). The sensitivity threshold (Limit of Detection - LOD) for variant detection was set at ≤0.5 %.

**Results:**

Twenty-one samples (12.4 %) did not meet the quality criteria for *ESR1* analysis. *ESR1* mutations were identified in 29.1 % (n = 41) of the remaining 141 cases. The most frequent *ESR1* variant was the p.Asp538Gly (53.7 %). Multiple *ESR1* mutations were observed in 29.3 % of mutated cases, and co-mutations were detected in 61 % of cases, mainly with *PIK3CA* (36.6 %) and *TP53* (12.2 %). The median variant allele frequency (VAF) of *ESR1* mutations was 1.46 %. No statistically significant difference in mutation frequency emerged between the two panels (p = 0.6993).

**Conclusions:**

*ESR1* mutations are detectable in approximately one-third of ER+/HER2− metastatic patients undergoing liquid biopsy. NGS platforms allow for sensitive and in-depth analysis, highlighting co-mutations of potential clinical and therapeutic relevance.

## 1 Introduction

Breast cancer (BC) is the most frequently diagnosed cancer in women worldwide and a leading cause of cancer-related death. About 70 % of invasive breast cancers express estrogen receptors (ERs), and these tumors are typically treated with endocrine therapies that modulate or deprive the BC of the hormones. However, a significant proportion of patients develop secondary resistance to hormonal treatments, particularly being identified as an important molecular mechanism of acquired resistance in hormone receptor-positive/human epidermal growth factor receptor 2-negative breast cancer (HR+/HER2- BC) patients after the first line of endocrine therapy (ET) [[1], [2], [3], [4], [5], [6]]. These mutations, which are uncommon in primary tumors but enriched following aromatase inhibitor exposure, confer ligand-independent activity and drive disease progression [1,5,7,8].

In this scenario, liquid biopsy emerged as the most promising biological source for recovering nucleic acids to test *ESR1* in the diagnostic routine practice of HR+/HER2- BC patients. In fact, the analysis of *ESR1* mutations through liquid biopsy, by measuring circulating tumor DNA (ctDNA), has gained clinical relevance as a non-invasive approach to molecularly monitor patients with metastatic breast cancer (mBC) [6,[9], [10], [11]].

The clinical importance of detecting *ESR1* mutations in liquid biopsies was validated in two significant prospective studies (see Table 1). The EMERALD study (NCT03778931) was a phase III randomized trial that evaluated the efficacy of elacestrant, an oral selective estrogen receptor degrader (SERD), in patients with ER+/HER2− mBCs who had been treated with at least one line of endocrine therapy and a CDK4/6 inhibitor. The study revealed that elacestrant significantly improved progression-free survival (PFS) in patients with *ESR1* mutations detected in circulating tumor DNA (ctDNA) compared to standard endocrine therapy - fulvestrant or aromatase inhibitor (AI), reducing the risk of progression by 45 % (HR 0.55, p = 0.0005) [[12], [13], [14]].

**Table 1:** **Key features of the EMERALD and PADA-1 clinical trials**. mBC: metastatic breast cancer; ER: estrogen receptors; HER2: human epidermal growth factor receptor 2; AI: aromatase inhibitors; SERD: selective estrogen receptor degrader; PFS: progression-free survival.

Study	Type of study	Cohort	Therapy	Comparison	Primary Endpoint	Outcome
**EMERALD**	Phase III, randomized	mBC ER+/HER2− preatreated with AI + CDK4/6i	Elacestrant (oral, SERD)	Standard endocrine therapy	PFS	Improved PFS (HR 0.55; p = 0.0005)
**PADA-1**	Phase III, randomized	mBC ER+/HER2− in 1st line with palbociclib + letrozole	Early switch to fulvestrant + palbociclib in case of emerging ESR1 mutation	AI + palbociclib continuation	PFS	Significantly prolonged PFS in the “switched” arm

Concurrently, the PADA-1 trial (NCT03079011), a phase III randomized study conducted in patients with ER+/HER2− mBC treated with palbociclib (CDK4/6 inhibitor) and letrozole (AI), revealed that identification of emerging *ESR1* mutations in ctDNA early on (in the absence of radiological progression) and subsequent replacement of letrozole with fulvestrant led to significant prolongation of PFS compared to continuing the initial therapy [15,16].

Other clinical studies have highlighted the presence of *ESR1* mutations in the ctDNA of patients previously treated with aromatase inhibitors, with a prevalence ranging from 20 % to 40 % [17,18].

Mutations in the *ESR1* gene in mBC can be detected through the analysis of ctDNA extracted from plasma. Commonly used methodologies include highly sensitive technologies, such as digital PCR (dPCR) and next-generation sequencing (NGS), that are capable of identifying low-frequency variants (<1 %) [10,19]. dPCR can quantify known hotspot mutations (e.g., p.Tyr537Ser, p.Asp538Gly), while NGS provides broader *ESR1* exons coverage, facilitating the identification of less common variants and of co-mutations [6,9,20]. Although singleplex platforms can be routinely adopted for *ESR1* mutation detection, the heterogeneous distribution of *ESR1* activating mutations and the large number of predictive biomarkers approved for clinical practice favor the utilization of NGS platforms [6,11,21].

Given this evidence, molecular characterization of the *ESR1* gene through liquid biopsy is a promising approach for personalizing therapy in patients with advanced breast cancer. In this study, we analyze frequency, distribution, and clinical impact of *ESR1* mutations detected in ctDNA from a large cohort of mBC patients to explore their potential as predictive and prognostic biomarkers in the clinical setting.

The aim of this study is to demonstrate the experience of an Italian referral center in analyzing *ESR1* in a cohort of ER+/HER2− BCs using two different NGS panels.

## 2 Material and methods

A total of 161 samples from patients whose breast cancer progressed after treatment with endocrine therapy and a cyclin inhibitor were analyzed in one year at the Molecular Pathology Laboratory of Solid Tumor – IRCCS Policlinico di S.Orsola. At least 7.5 ml of peripheral blood was obtained from each patient and transferred into an EDTA tube or a ctDNA preservation tube. EDTA tubes were processed in 2 h from the time of the blood sampling, and ctDNA preservation tubes within 24 h. Plasma was obtained after double centrifugation at 2000 g. The plasma was then stored at −80 °C or processed immediately for extraction [22]. Cell-free DNA (cfDNA) extraction was performed using the Maxwell® RSC cfDNA Plasma Kit (Promega, Italy). cfDNA analysis was performed using two different panels: The Oncomine™ Breast cfDNA Research Assay v2 (1.95 kb) (Thermo Fisher Scientific) (n = 102) and the Oncomine™ Precision Assay GX (14.15 kb) (Thermo Fisher Scientific) (n = 59). The first panel was run on a GeneStudio S5 Prime sequencer (Thermo Fisher Scientific) and the second panel on a Genexus instrument (Thermo Fisher Scientific). A list of the genes covered by the two panels is provided below.

Oncomine Breast cfDNA Assay v2 (Breast v2). Mutation in hotspot of the following genes: *AKT1, EGFR, ERBB2, ERBB3, ESR1, FBXW7, KRAS, PIK3CA, SF3B1, TP53* (CDS - coding sequence).

*Copy Number Variations (CNVs)*: *CCND1, ERBB2, FGFR1*.

Oncomine Precision Assay (OPA). Mutation in hotspot of the following genes: *AKT1, AKT2, ALK, AR, ARAF, BRAF, CDK4, CDKN2A, CHEK2, CTNNB1, EGFR, ERBB2, ERBB3, ERBB4, ESR1, FGFR1, FGFR2, FGFR3, FGFR4, FLT3, GNA11, GNAQ, GNAS, HRAS, IDH1, IDH2, KIT, KRAS, MAP2K1, MAP2K2, MET, MTOR, NRAS, NTRK1, NTRK2, NTRK3, PDGFRA, PIK3CA, PTEN, RAF1, RET, ROS1, SMO, TP53*.

*Gene rearrangements*. *ALK, BRAF, ESR1, FGFR1, FGFR2, FGFR3, MET, NRG, NTRK1, NTRK2, NTRK3, NUTM1, RET, ROS1, RSPO2, RSPO3*.

*Copy Number Variations (CNVs)*: *ALK, AR, CD274, CDKN2A, EGFR, ERBB2, ERBB3, FGFR1, FGFR2, FGFR3, KRAS, MET, PIK3CA, PTEN*.

The analysis was conducted using Ion Reporter 5.20 software for samples analyzed with the Breast v2 panel and Genexus software for samples analyzed with the OPA panel. Samples were considered suitable for *ESR1* analysis if the Limit of Detection (LOD) was equal to or less than 0.5 % [23]. All data used in the present study were completely anonymized.

## 3 Results

**Fig. 1:**
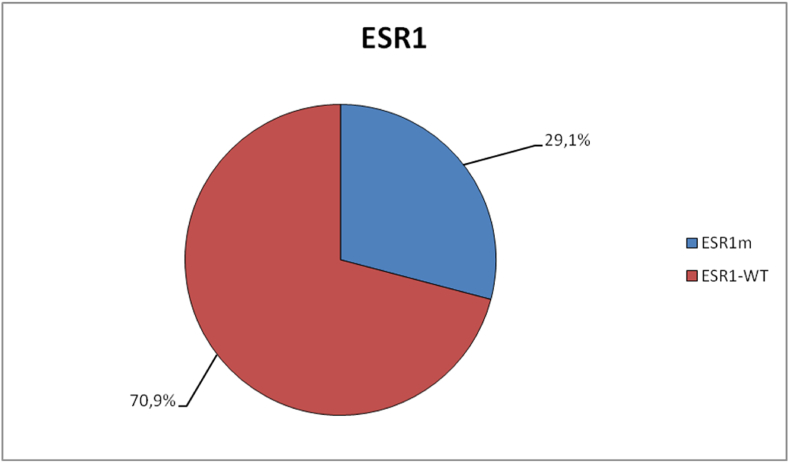
**Frequency of *ESR1* mutations in the cohort analyzed.** ESR1m: *ESR1* mutation; ESR1-WT: *ESR1* wild-type.

Of the 161 cases analyzed, 20 (12.4 %) had an inadequate LOD (>0.5 %) for *ESR1* analysis. Ten out of 102 (9.8 %) cases were found to be inadequate using the Breast v2 panel, while 10 out of 59 (17 %) cases were inadequate using the OPA panel. As expected, given the OPA panel's larger size, there were more non-informative cases than with a smaller, targeted panel. However, the difference was small and not statistically significant. Of the remaining 141 cases, 41 (29.1 %) had an *ESR1* mutation (ESR1m) (Fig. 1). Of those, 28 (30.4 %) were detected using the Breast v2 panel, and 13 (26.5 %) were detected using the OPA panel, a difference that was not statistically significant (p = 0.6993, Fisher's exact test).

**Fig. 2:**
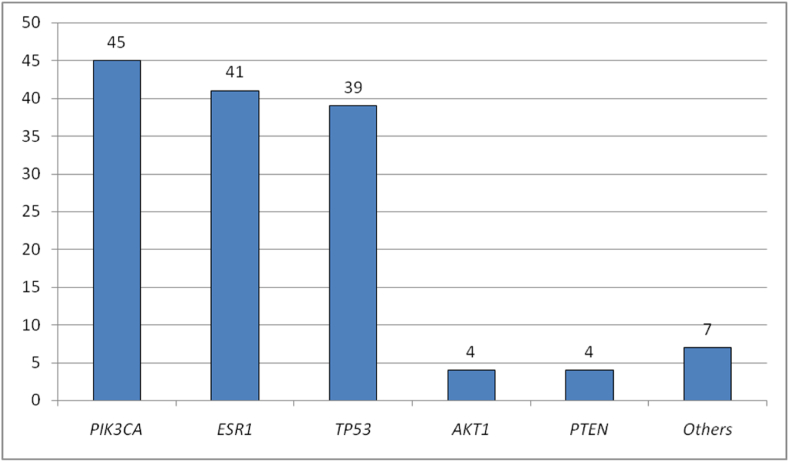
Distribution of mutations identified in the ctDNA of the samples analyzed.

Among the 141 cases with adequate LOD, *PIK3CA* was the most frequently mutated gene, detected in 45 cases (31.9 %). *PIK3CA* was followed by *ESR1*, which occurred in 41 cases (29.1 %), and *TP53*, which occurred in 39 cases (27.7 %). *AKT1* variants were found in four cases (2.8 %), as were *PTEN* variants. Variants in other genes were identified in seven cases (5.0 %) (Fig. 2).

The most frequently identified *ESR1* variant was the p.(Asp538Gly), followed by the p.(Tyr537Asn) and p.(Glu380Gln). Table 2 shows the frequencies of the various variants among the 41 ESR1m cases.

**Table 2:** **Types of *ESR1* variants identified in the cohort analyzed.** ESR1m: *ESR1* mutation.

*ESR1* variant – protein level	*ESR1* variant – DNA level	#cases (% of 41 ESR1m)
p.(Asp538Gly) (p.D538G)	c.1613A > G	22 (53.7)
p.(Tyr537Asn) (p.Y537N)	c.1609T > A	14 (34.1)
p.(Glu380Gln) (p.E380Q)	c.1138G > C	12 (29.3)
p.(Tyr537Cys) (p.Y537C)	c.1610A > G	8 (19.5)
p.(Tyr537Ser) (p.Y537S)	c.1610A > C	8 (19.5)
p.(Leu536His) (p.L536H)	c.1607_1608delinsAT	1 (2.4)
p.(Arg555Cys) (p.R555C)	c.1663C > T	1 (2.4)

Of the 41 ESR1m cases, in 16 (39 %) only one *ESR1* variant was detected, while 12 cases (29.3 %) harbored multiple *ESR1* mutations. The remaining 25 cases (61.0 %) had mutations in other genes in addition to those in *ESR1* (Table 3). The most frequent combination was *ESR1* and *PIK3CA* (in 15 of the 41 ESR1m cases) (Table 3). In five cases, three concomitant variants were detected in the *ESR1-PIK3CA-TP53* genes (Table 3). Overall, 25 of the 141 (17.7 %) evaluable samples harbored a concomitant mutation together with the one in *ESR1* (Table 3).

**Table 3:** **Presence of *ESR1* co-mutation with other genes.** ESR1m: *ESR1* mutation.

Gene(s)	Sample (% of 41 ESR1m samples)
*ESR1* only	16 (39.0)
*ESR1+PIK3CA*	15 (36.6)
*ESR1+PIK3CA + TP53*	5 (12.2)
*ESR1+TP53*	2 (4.9)
*ESR1+PTEN*	2 (4.9)
*ESR1+AKT1*	1 (2.4)

**Fig. 3:**
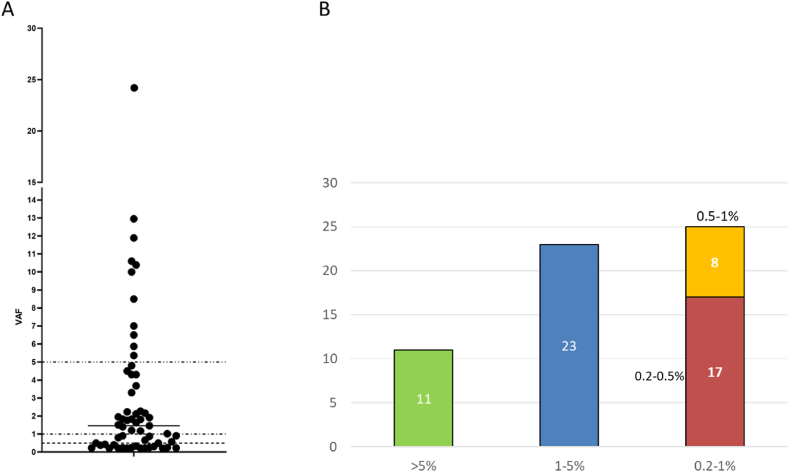
**A) Dot-plot of VAF distribution of *ESR1* variants.** The dotted lines indicate 5 %, 1 %, and 0.5 % of VAF. **B) Number of cases grouped according to ESR1m VAF range**.

The median VAF of *ESR1* variants was 1.46 % (mean 3.01 %, ranging from 0.21 to 24.2 %) (Fig. 3A). Of these, 81.4 % had a VAF of less than 5 %, 42.4 % had a VAF of less than 1 %, and 32.2 % had a VAF of less than 0.5 % (Fig. 3B).

Of the 100 *ESR1*-wild-type cases, 48 (48.0 %) showed no mutations, while at least one mutation, different from *ESR1*, was identified in the other 52 cases (52.0 %) (Table 4). The more common mutations identified were *PIK3CA* (25 cases) and *TP53* (32 cases). Other variants were identified in the following genes: *AKT1* (3 samples), *SF3B1* (1 sample), *BRAF* (2 samples), *ERBB2* (2 samples), *PTEN* (3 samples), and *KRAS* (2 samples). Seventeen samples harbored more than one mutation (Table 4).

**Table 4:** **Molecular status of ESR1-WT samples.** WT: wild-type.

Gene	Samples (% of 141 cases)
WT	48 (34.0)
*TP53*	16 (11.3)
*PIK3CA*	12 (8.5)
*PIK3CA + TP53*	12 (8.5)
*AKT1*	3 (2.1)
*PTEN*	2 (1.4)
*PIK3CA + SF3B1*	1 (0.7)
*TP53+BRAF*	1 (0.7)
*TP53+ERBB2*	1 (0.7)
*TP53+BRAF + PTEN*	1 (0.7)
*TP53+KRAS*	1 (0.7)
*KRAS*	1 (0.7)
*ERBB2*	1 (0.7)

## 4 Discussion

The detection of *ESR1* mutations in ctDNA through liquid biopsy allows the molecular evaluation of the tumor using a minimally invasive alternative to solid tissue-based genotyping [6,8,19,[24], [25], [26], [27], [28]]. Advances in molecular techniques (such as dPCR and NGS) have substantially improved the sensitivity of *ESR1* detection in plasma, allowing the identification of hotspot alterations at mutant allele frequencies below 0.5 % [19,24,26,27]. These technologies, applied to the liquid biopsy matrix, also facilitate longitudinal assessment of clonal evolution and early identification of resistance, supporting timely modification of endocrine treatment [6,18,19,25,26]. Several studies confirm that liquid biopsy-based *ESR1* testing is concordant with tissue genotyping and is associated with clinical outcomes, including PFS on AI-based therapies [5,7,8,18,28]. The American Society of Clinical Oncology recognizes the potential of *ESR1* mutation analysis in ctDNA to guide systemic therapy, although the evolution of clinical implementation depends on further validation from ongoing trials [7]. Liquid biopsy-based *ESR1* mutation detection represents a pivotal advance in precision oncology for breast cancer, enabling personalized management and improved patient outcomes [6,8,18,25,28,29].

Our analysis of a cohort of 161 patients with ER+/HER2− metastatic breast cancer confirms the relevance of *ESR1* gene mutation detection, which was identified in approximately 30 % of eligible cases. Our results are consistent with those reported in the scientific literature, where the prevalence of *ESR1* mutations in ctDNA ranges from 20 % to 40 % in patients who have previously been treated with aromatase inhibitors [1]. The distribution of variants, dominated by *ESR1* p.Asp538Gly and p.Tyr537Asn, is also consistent with what observed in large-scale cohort studies [1,6].

One particularly relevant finding in our study is the high frequency of co-mutations, particularly those involving *PIK3CA* and *TP53* genes. The detection of co-mutations in genes such as *PIK3CA* and *TP53* in over 60 % of ESR1-mutated cases suggests the presence of oncogenic cooperation of multiple tumor subclones. These factors may influence therapeutic efficacy and the selection of targeted treatments [14,16,30,31]. For example, the concurrent presence of *PIK3CA* and *ESR1* mutations, observed in approximately one-third of our cases, has been shown to have significant clinical implications regarding the response to PI3K inhibitors and SERDs [[12], [13], [14]]. This suggests that, in addition to developing resistance to hormone therapy, tumors of ER+/HER2− patients with metastatic disease exhibit complex clonal evolution, which may require combined therapeutic strategies. For example, the simultaneous presence of alterations in *ESR1* and *PIK3CA* could make a combination of PI3K inhibitors and new oral SERDs a potentially useful treatment option.

Although liquid biopsy does not suffer from the pre-analytical problems typically associated with the analysis of routinely processed solid tissue samples (e.g., degradation due to formalin fixation), it is not without its own issues, and workflow must be carefully planned. For example, when using EDTA tubes for blood collection, plasma separation must occur within 2 h. In our study, samples from three patients were processed in parallel within 2 h of collection and after storage at room temperature for three additional hours before plasma separation. The LODs obtained from the samples processed within 2 h were 0.25 %, 0.35 %, and 0.38 %. On the other hand, the LODs of the three samples left at room temperature for a total of approximately 5 h after collection of peripheral blood were 0.45 %, 0.75 %, and 0.88 %, respectively (data not shown). These data emphasize the importance of following standardized and stringent operating protocols, in line with international guidelines [32]. Moreover, standardization of molecular pathology workflows and reporting is essential to ensure diagnostic reproducibility for clinical decision making [25].

From a technical point of view, the use of two NGS panels allowed us to compare the analytical performance of different NGS assays. Although the Oncomine Precision Assay panel has greater gene coverage and potentially less depth per target, there were no significant differences in its ability to detect *ESR1* mutations compared to the Breast cfDNA v2 panel, which is more focused. These results suggest that both platforms can be adopted in clinical settings under the same operating conditions, with the choice guided by laboratory availability and the extent of the mutational profile analysis required for clinical use.

The results of our study confirm that ctDNA analysis is a reliable and clinically useful tool to define the tumor molecular profile of ER+/HER2− patients with metastatic disease, which is essential to adequately treat those who have previously undergone endocrine therapy. Consistent with recent literature reports, approximately 30 % of our patients showed activating mutations in the *ESR1* gene.

Our study has some limitations. First, the lack of detailed clinical follow-up means that the mutations identified cannot be correlated with treatment response or survival data. Second, although there was no statistical difference in the results obtained with the Breast cfDNA Assay and Oncomine Precision Assay panels, the use of two different panels may represent a bias. Third, the clinical relevance of low-frequency *ESR1* mutations (VAF <0.5 %), which were detected in one-third of mutated cases in our study, requires further investigation.

In conclusion, our study strongly supports the case for routinely integrating liquid biopsies into the management of ER+/HER2− patients with metastatic disease. The identification of *ESR1* mutations and of their pertinent genomic co-alterations is a key step towards making precision medicine applicable in clinical practice.

## Ethical approval/patient consent

The study was approved by the Independent Ethics Committee "Comitato Etico di Area Vasta Emilia Centro”, number of study 258/2024/Sper/AOUBo.

## Declaration of competing interest

The authors declare the following financial interests/personal relationships which may be considered as potential competing interests: Dario de Biase reports a relationship with Menarini Stemline Italia SrL that includes: speaking and lecture fees. Dario de Biase reports a relationship with GSK that includes: speaking and lecture fees. Dario de Biase reports a relationship with Eli Lilly that includes: speaking and lecture fees. Dario de Biase reports a relationship with AstraZeneca that includes: speaking and lecture fees. If there are other authors, they declare that they have no known competing financial interests or personal relationships that could have appeared to influence the work reported in this paper.
